# The effectiveness of workplace health promotion programs on self-perceived health of employees with a low socioeconomic position: An individual participant data meta-analysis

**DOI:** 10.1016/j.ssmph.2021.100743

**Published:** 2021-01-26

**Authors:** Hanneke van Heijster, Cécile R.L. Boot, Suzan J.W. Robroek, Karen Oude Hengel, Jantien van Berkel, Emely de Vet, Pieter Coenen

**Affiliations:** a Department of Social Sciences, Chair Group Consumption & Healthy Lifestyles, Wageningen University & Research, Hollandseweg 1, 6706 KN, Wageningen, the Netherlands; b Department of Public and Occupational Health, Amsterdam Public Health Research Institute, Amsterdam UMC, Vrije Universiteit Amsterdam, Amsterdam, the Netherlands; c Erasmus University Medical Center, Department of Public Health, P.O. Box 2040, 3000 CA, Rotterdam, the Netherlands; d Netherlands Organisation for Applied Scientific Research TNO, Department of Healthy Living, Schipholweg 79-86, 2316 ZL, Leiden, the Netherlands

**Keywords:** Workplace health promotion, Employees, Low socioeconomic position, Self-perceived health

## Abstract

The aim of the current study was to evaluate whether workplace health promotion programs improve self-perceived health of employees with a low socioeconomic position (SEP), and whether differential effects exist between individuals with a low SEP for gender, marital status or age. Individual participant data from six Dutch intervention studies aiming at promoting healthy behavior and preventing obesity in the work setting, with a total of 1906 participants, were used. The overall intervention effect and interaction effects for gender, marital status and age were evaluated using two-stage meta-analyses with linear mixed regression models. In the first stage effect sizes of each study were estimated, which were pooled in the second stage. Compared to control conditions, workplace health promotion programs did not show an overall improvement in self-perceived health of employees with a low SEP (β0.03 (95%CI: −0.03 to 0.09)). Effects did not differ across gender, marital status and age. Future research could be focused on the determinants of self-perceived health next to health behavior to improve the health of employees with a low SEP.

## Introduction

Individuals with a low socioeconomic position (SEP) generally have a poorer health than individuals with a high SEP (Mackenbach et al., 2008). Individuals with a low SEP have a shorter life-expectancy and live on average shorter in good health (Beckfield et al., 2013; Cutler et al., 2006; Kunst et al., 2005). In Europe, differences in life-expectancy of more than 10 years between SEP groups within countries have been observed. Moreover, the observed difference in *healthy* life-expectancy between these groups even ranges from 10 to 23.1 years (OECD, 2016). Health inequalities are also reflected in poorer self-perceived health in individuals with a low SEP compared to individuals with a high SEP (Eurostat, 2018), and self-perceived health is an important predictor of future morbidity and mortality (Reile et al., 2017). Workplace health promotion programs aiming to promote healthy behavior are considered promising to improve self-perceived health of employees with a low SEP, as poorer health behavior, which is more common in individuals with a low SEP, is a known risk factor of a poor self-perceived health (Goldstein et al., 1984; Johansson et al., 2019; Manderbacka et al., 1999; Shields and Shooshtari, 2001). However, it has rarely been evaluated to what extent workplace health promotion programs focusing on promoting healthy behavior actually do improve self-perceived health of employees with a low SEP.

Studies on the effects of workplace health promotion programs on health behaviors of employees with a low SEP show mixed results. Interventions that provide convenient access to programs can be effective for employees with a low SEP (Stiehl et al., 2018). For example, a workplace health promotion program in which fruit was offered for free at the workplace led to an increase in fruit and vegetable consumption in employees with a low SEP and their families on short-term (12 weeks) (Backman et al., 2011). Also, a systematic review showed that workplace health promotion programs focusing on physical activity can be modestly effective for employees with a low SEP (Cairns et al., 2015). However, a recent IPD meta-analysis showed no long-term (6–12 months) effects on behavioral outcomes such as physical activity, dietary behavior and smoking (Coenen et al., 2020, Robroek et al., 2020). It is unclear whether the in general modest and small effects on health behavior can still impact self-perceived health of employees with a low SEP. As combined improvements in health behavior have a larger effect on self-perceived health than improvements in single health behaviors (Harrington et al., 2010), modest improvements in various behaviors might still have an impact on self-perceived health. Next to that, workplace health promotion programs may also have an impact on other determinants of self-perceived health, for example when the programs improve social and emotional support or reduce distress (Kilpatrick et al., 2015; Shields and Shooshtari, 2001).

Some studies did report an improvement on self-perceived health from workplace health promotion programs focusing on health behavior. One meta-analysis showed modest improvements, although this was for employees in general, rather than for employees with a low SEP in particular (Rongen et al., 2013). Even though the attention in research on employees with a low SEP is growing, the group is still generally underrepresented (Stiehl et al., 2018), while they have a poorer health compared to employees with a higher SEP (Robroek et al., 2015). Therefore it often remains unclear whether employees with a low SEP profit from workplace health promotion programs. A recent study including cross-sectional data from nine European countries showed that participation in workplace health promotion programs is associated with a better self-perceived health in employees with a low SEP, if such programs consisted of a health check (van der Put et al., 2020). However, considering the cross-sectional character of the study, more research is needed to understand the causal relationship between workplace health promotion programs and self-perceived health of employees with a low SEP.

As the health potential is largest for employees with a low SEP, differences within the group of employees with a low SEP should be explored. Such within group differences in effectiveness are often not reported, while these insights are necessary to inform public policy and practice by formulating potential risk groups (Egan et al., 2009). First, differences may exist for employees with a low SEP at different ages, as a meta-analysis found that younger employees profit more from workplace health promotion programs than older employees (Rongen et al., 2013). Second, gender and marital status may influence effectiveness, as differences in participation in workplace health promotion programs have been reported for these factors (as well as for age) (Robroek et al., 2009). Female employees, married employees and younger employees are more likely to participate in workplace health promotion programs. Although participation does not directly reflect differences in effectiveness, the extent to which certain groups are willing to participate in an intervention may influence the potential intervention impact. One of the facilitators to have a positive intention towards participation in workplace health promotion programs is thinking that participation is useful (Rongen et al., 2014). Groups that are generally more likely to participate in programs may profit more from programs because of the higher expectations they may have had about the usefulness of participation from the start.

The aim of this study is to evaluate whether workplace health promotion programs improve self-perceived health of employees with a low SEP, and whether differential effects can be found within employees with a low SEP regarding gender, marital status and age. As employees with a low SEP are generally underrepresented in research in the field of workplace health promotion (Stiehl et al., 2018) while they have a poorer health compared to employees with a higher SEP (Robroek et al., 2015) the focus of this paper is on employees with a low SEP. For the analysis, data from a large dataset with individual participant data (IPD) on Dutch workplace health promotion programs will be used (Oude Hengel et al., 2019). Using IPD has three advantages compared to data from a conventional meta-analysis. First, the availability of raw data in IPD allows to focus specifically on employees with a low SEP, by selecting out their data from the original studies. This is usually not possible in a conventional meta-analysis, because effects are only available on a group level, which often comprises employees in general. Second, an IPD meta-analysis allows to perform analyses that were not performed in the original articles, like the analyses of subgroup differences in effectiveness for gender, age and marital status. Lastly, IPD allows to report the effects on self-perceived health, an outcome that is often evaluated in the original studies as a secondary outcome, but is generally not reported in publications. Because of the strong predictive value for future morbidity and mortality of self-perceived health (Reile et al., 2017), it was considered relevant to evaluate the effects on this outcome.

## Methods

### Search strategy and selection of studies

The current paper was prepared in accordance with the PRISMA-IPD guidelines (Stewart et al., 2015). Details of the composition of the IPD dataset were reported in the published protocol (Oude Hengel et al., 2019), which was registered in Prospero (register number: CRD42018099878).

A systematic approach was used to identify relevant studies aimed at workplace health promotion of health behavior and prevention of obesity. The search was performed in February 2018. Search terms were related to: health behavior, obesity, intervention, evaluation, and worker/workplace. For published studies, the following electronic databases were used: Embase, Medline Ovid, Web of Science, Cochrane Central and Google scholar. In addition, reference lists of relevant reviews and meta-analysis were checked. For unpublished studies, trial registers, databases of major Dutch funding agencies and the Dutch database for lifestyle interventions were checked. Only studies performed in the Netherlands were included, because of the occupational setting of the Netherlands in which employers are responsible for sickness benefits during the first two years of sickness absence. Including trial data from the same occupational setting allowed to make a fair comparison.

Inclusion criteria on the study level were: 1) a preventive intervention study aiming to promote health behavior and/or prevent obesity, 2) targeting at workers, 3) performed in the Netherlands, 4) from a study design with at least a pre- and post-measurement and a comparative reference group, 5) presents an indicator for SEP (e.g. educational level, job title or income). No restrictions in terms of year of publication were applied in the searches. Two independent reviewers (PC and SR) screened all records for eligibility in April and May 2018. In case of disagreement, consensus was reached in a meeting or by consulting a third reviewer (KOH). A total of 34 studies (with 88 articles) on health promotion programs were considered eligible for the dataset.

For the current meta-analysis, only studies measuring self-perceived health and with data on employees with a low SEP were used. Meta-analyses on body mass index (Robroek et al., 2020) and lifestyle outcomes (Coenen et al., 2020) were reported elsewhere.

#### Data extraction and methodological quality

The principle researcher of each eligible study was contacted and asked to share the original individual participant study data. If the researchers agreed with sharing the data, they were asked to sign a data transfer agreement and to transfer their data, code books and syntaxes. All study data were harmonized using definitions of each of the variables as formulated by the research team and described in a code book.

On a study level the following data were extracted from the eligible studies: study design, content and setting, and primary and secondary outcomes including measurement method. On a participant level, the indicator measured for SEP and characteristics of the participants such as gender, age and marital status were extracted. To evaluate the methodological quality of the selected studies, a modified version (Rongen et al., 2014) of the Cochrane risk of bias tool (Higgins and Green, 2011) was used, consisting of nine criteria regarding randomization, blinding, similarity of groups, compliance, loss to follow-up, intention-to-treat, confounder adjustment, data collection methods, and follow-up duration.

#### Data harmonization

##### SEP

In most of the studies used in this meta-analysis, SEP was based on educational level. SEP was defined as low when the participants had a low educational level according to the 1997 International Standard Classification of Education (ISCED-97) (no education, primary school or lower vocational education). In one study among construction workers (Groeneveld et al., 2010), no information was available on educational level. In that study, occupational class was used to define SEP, with blue collar workers being categorized as low SEP.

##### Self-perceived health

All studies measured self-perceived health with the first question of the RAND-36 item Health Survey (Sanderman, 2012), using the cluster ‘general health perception’. This cluster consists of the following question: ‘Overall, how would you rate your health?’ with answer options a 5-point scale. Five studies used the US version of this scale (Jürges et al., 2008), with different labels ranging from ‘poor’ (1) to ‘excellent’ (5), whereas one study (Kouwenhoven-Pasmooij et al., 2018) used the WHO version consisting of answer categories ranging from ‘very bad’ (1) to ‘very good’ (5). The labels were not recoded as doing so would have made no difference because a two-stage meta-analysis approach was used in this study (see statistical analysis for more details). Self-perceived health was a primary outcome in one study (Kouwenhoven-Pasmooij et al., 2018). In the other five studies self-perceived health was a secondary outcome. For statistical analyses self-perceived health was treated as a continuous variable, to enable to detect subtle improvements in self-perceived health.

Self-perceived health was measured at three moments: at baseline, directly after the intervention (immediate effects) and after the end of the intervention (sustained effects). Since the duration of interventions and follow-up duration differed between studies, immediate and sustained effects did not have the same absolute definition for all studies. In five out of six studies immediate effects were measured 6 months after baseline, while in one study the measurement of immediate effects took place after 24 months. In four out of six studies the measurement of sustained effects took place 12 months after baseline, and in two of the studies 24 months after baseline.

##### Gender, marital status, age

Gender was considered a dichotomous (men/women) variable. Marital status was harmonized into a dichotomous variable with outcome categories married/living together and single (i.e. being single, divorced, widow/widower, and being in a relationship but not living together). Age was considered a continuous variable in order to minimize the loss of data due to an insufficient number of data points in the age categories.

#### Statistical analysis

A two-stage meta-analysis with linear mixed modelling was performed to study the effectiveness of the interventions on self-perceived health of employees with a low SEP, and to evaluate differential effects for subgroups for gender, age and marital status. In the first stage, for each study an effect size was estimated for the employees with a low SEP in that study. Also, the interaction effects for gender, age and marital status were assessed in each study within the group of employees with a low SEP. In the second stage, the overall effect sizes and interaction effects of the individual studies were pooled using the Stata *admetan* (Version 14) function. A study was only included in the statistical analysis when at least 20 data points of participants with a low SEP were available. Interaction effects were only estimated for those studies with a least 20 data points in each subgroup for which a differential effect was evaluated (for example group ‘men’ and group ‘women’). If there were less than 20 data points, the study was excluded for the specific analysis.

Because no statistically significant difference was found between immediate effects (immediately after the intervention) and sustained effects (after the end of the intervention), both time points were added jointly to the model, and a random intercept for participant was added. As such, both intermediate and sustained effects were considered comparable and were statistically treated as such in the statistical model. This procedure enhances statistical power, while acknowledging the within participant dependence using random intercepts for participants. All models were adjusted for baseline values of the outcome. The model for the overall effect was also adjusted for age, gender and marital status, because all three are associated with self-perceived health (Shields and Shooshtari, 2001).

For cluster randomized controlled trials, intra-class correlation coefficients (ICCs) were estimated to evaluate the variance within and between clusters. As no ICC values > 0.10 were found, random intercept for clustering was not added to the model. As described in the protocol paper, heterogeneity among studies was assessed in a sensitivity analysis in which each of the studies were subsequently left out of the analysis, assessing its impact on the effect size. For statistical analyses Stata (version14) was used, and Review Manager (version 5.3.5) was used for making forest plots to illustrate individual study effect sizes. In all analyses, the level of statistical significance was set at p < 0.05.

## Results

### Study selection

**Fig. 1:**
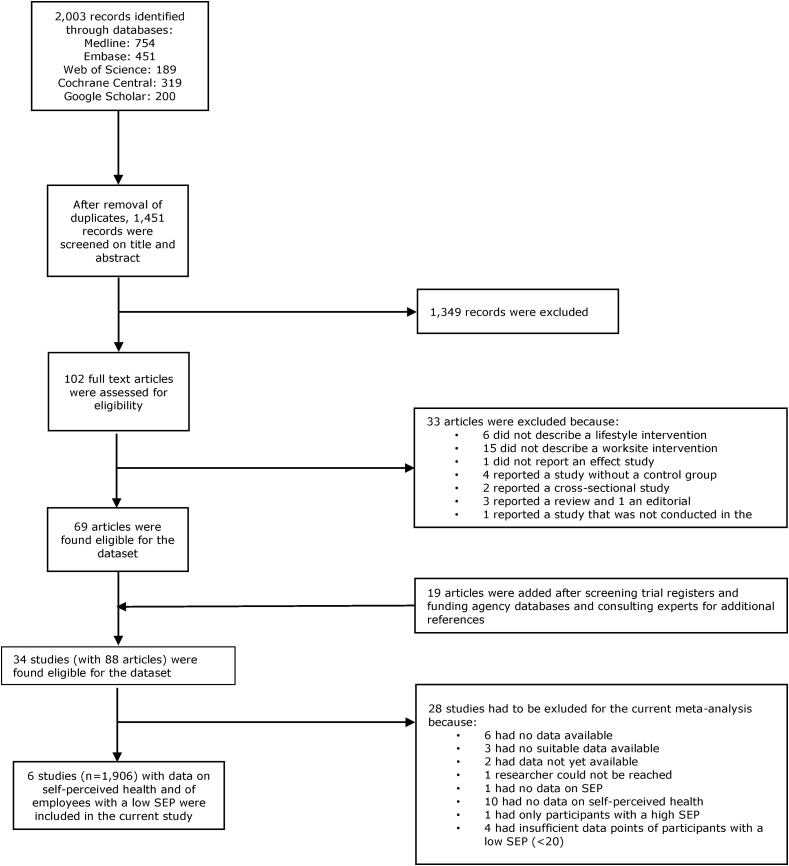
Flow chart of describing the study inclusion process.

Of the 34 studies that were found eligible for the database, 6 were included in the current IPD meta-analysis (Fig. 1), which focused on employees with a low SEP. 28 studies were excluded because data were not available anymore (n = 6), no IPD were available (n = 3), data was not available yet (n = 2), the researchers could not be reached (n = 1), no indication of SEP was available (n = 1), no information on self-perceived health was available (n = 10), there were no participants with a low SEP (n = 1), there were not enough participants with a low SEP (<20) (n = 4). Data from 6 studies in which employees with a low SEP participated (n = 1906) were used for the current meta-analysis (Table 1). In these studies, the number of participants with a low SEP ranged from 66 to 990 per study, with a median of 381.

**Table 1:** Main characteristics of the studies included in this meta-analysis.

Author (year)	N^a^ (low SEP) (% of all participants in study)	Organizational context	Study population characteristics						Targeted behavior	Intervention components
			Gender		Marital status		Age (Mean (SD))	% good or better health on baseline		
			Male (N, %)	Female (N,%)	Married/living together (N, %)	Single (N, %)				
Viester et al. (2018)	361 (68%)	Construction	361 (100%)^b^	0 (0%)^b^	300 (83%)	39 (13%)	47.9 (8.8)	93.2	Physical activity and healthy diet	Counselling
Van Wier et al. (2011)	66 (5%)	Various (IT, hospital, insurance, financial, police)	43 (65%)	23 (35%)	54 (93%)^b^	12 (7%)^b^	47.5 (7.6)	90.0	Physical activity and healthy diet	Counselling
Strijk et al. (2012)	108 (10%)	Hospital	21 (19%)	87 (81%)	80 (74%)	28 (26%)	54.6 (4.8)	90.9	Physical activity, healthy diet and relaxation	Combined and environmental component
Groeneveld et al. (2010)	990 (75%)	Construction	990 (100%)^b^	0 (0%)^b^	814 (82%)	139 (14%)	47.0 (8.9)	60.4	Physical activity, diet and smoking cessation	Counselling
Robroek et al. (2012)	247 (21%)	Various (health care, commercial services, executive branch of government)	145 (59%)	102 (41%)	191 (77%)	56 (23%)	47.3 (8.3)	90.7	Physical activity and healthy diet	Counselling
Kouwenhoven-Pasmooij et al. (2018)	134 (20%)	Military, police, hospital	117 (87%)^b^	17 (13%)^b^	124 (81.8%)^b^	10 (18.2%)^b^	52.0 (5.2)	72.4	Various lifestyle behaviors, based on risk profile participant	Counselling

a = concerns the number of participants with self-perceived health information from baseline and follow-up measurement(s).

b = excluded from subgroup analysis for either gender and/or marital status because there were not enough data points (<20 participants) in one of the subgroups.

### Study characteristics

Across the six included studies, the majority of the participants were men (88%), married or living together (82%) and had an average age of 48.0 (SD: 8.6) years. In two of the six studies, both in the construction industry, only men participated (Groeneveld et al., 2010; Viester et al., 2018), whereas in the remaining four studies both men and women participated. One of these studies was conducted in a hospital setting (Strijk et al., 2012) and three studies were conducted in various occupational settings, including IT, health care, commercial/financial, police, and governmental settings (Kouwenhoven-Pasmooij et al., 2018; Robroek et al., 2012; Van Wier et al., 2011).

Five out of six studies focused on workers that were defined as a risk group (indicated prevention), only including employees with higher risk on cardiovascular disease (Groeneveld et al., 2010; Kouwenhoven-Pasmooij et al., 2018), workers with overweight (Van Wier et al., 2011; Viester et al., 2018) or older workers (Strijk et al., 2012). Five studies aimed to promote physical activity and a healthy diet (Groeneveld et al., 2010; Robroek et al., 2012; Strijk et al., 2012; Van Wier et al., 2011; Viester et al., 2018), of which two also targeted other behaviors such as smoking cessation (Groeneveld et al., 2010) and relaxation (Strijk et al., 2012). One study focused on various health behaviors depending on the risk profile of the participant (Kouwenhoven-Pasmooij et al., 2018). All studies included a counselling component (e.g. coaching sessions, personalized feedback), and one study also included an environmental component consisting of offering fruit at the workplace (Strijk et al., 2012). The average self-perceived health at baseline was 3.03 (SD: 0.73).

### Overall effect

**Fig. 2:**
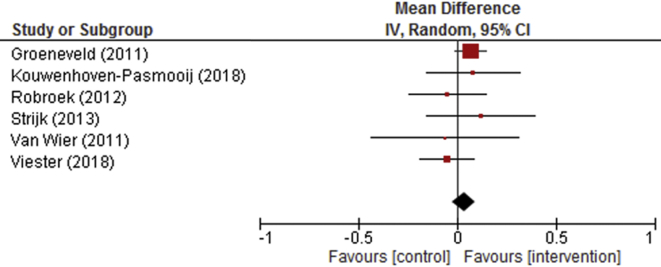
Forest plot depicting the individual and pooled study effects of the health promotion programs on self-perceived health.

Overall, no significant effects were found for self-perceived health among workers with a low SEP in the intervention groups compared to the control groups (beta: 0.03 on a 5-point scale (95%CI: −0.03 to 0.09)) (Table 2). Moreover, none of the six underlying studies showed a statistically significant increase in self-perceived health in the intervention groups compared to the control groups (Fig. 2). The sensitivity analysis showed consistency across studies.

**Table 2:** Findings regarding the effectiveness on self-perceived health. Overall effects and interaction effects for gender, marital status and age are presented. Effects sizes are expressed in betas with 95% confidence intervals (95% CI).

	Studies N	Participants n	Effects on self-perceived health beta [95% CI]
**Overall**	6	1906	0.03 [-0.03 0.09]
**Gender (interaction)**			
Women vs. men	3	421	0.13 [-0.18 0.44]
**Marital status (interaction)**			
Single vs. married/living together	4	1647	0.08 [-0.11 0.26]
**Age (interaction)**	6	1906	0.00 [-0.02 0.01]

### Subgroup analyses

Because of an insufficient number of participants (<20) in each stratum, studies had to be excluded when performing the subgroup analysis for gender and marital status (Table 1). For gender, three studies could be used for subgroup analysis (Robroek et al., 2012; Strijk et al., 2012; Van Wier et al., 2011), with 421 participants in total. For marital status, four studies could be used (Groeneveld et al., 2010; Robroek et al., 2012; Strijk et al., 2012; Viester et al., 2018) with 1647 participants in total.

No significant interaction effects were found for gender β 0.13 [95% CI: −0.18 to 0.44], marital status β 0.08 [95% CI: −0.11 to 0.26], or age β 0.00 [95% CI: −0.01 to 0.01].

## Discussion

The aim of this study was to evaluate whether workplace health promotion program focused on promoting health behavior improve self-perceived health of employees with a low SEP and whether differential effects exist within this group for age, gender and marital status. The results show that workplace health promotion programs described in this study did not improve self-perceived health of employees with a low SEP, neither did these interventions have differential effects for subgroups of gender, marital status and age.

The expectations with regard to effectiveness of workplace health promotion program focusing on health behavior on self-perceived health were mixed. Some studies showed positive effects on health behavior of employees with a low SEP (Backman et al., 2011; Cairns et al., 2015; Stiehl et al., 2018), but these effects were generally modest. Previous research did show modest effects of workplace health promotion programs focused on health behavior on self-perceived health for employees in general, such as one meta-analysis among eighteen studies (Rongen, 2013). The current meta-analysis did not provide evidence for this effect for employees with a low SEP in particular. In both the current study as the study of Rongen and colleagues, the workplace health promotion programs often contained a counselling component, but in the latter mainly white collar employees participated. Possibly, individual level components such as counselling are less suitable for employees with a low SEP (Beauchamp et al., 2014; Magnée et al., 2013). This may partly explain why the results from this study differ from previous research on the effects of workplace health promotion programs on self-perceived health.

However, a factor that should be considered in the lack of effect, is the relatively high baseline scores of self-perceived health of the study samples (Table 1) compared to the average Dutch lower SEP population. In 2017, 58.4% of the Dutch individuals with a low SEP (based on educational level), perceived their health as ‘good’ or ‘very good’ (CBS, 2018). In the current study, this percentage was 80.2%, being considerably higher than the country average (Table 1). This can possibly be explained by the fact that individuals with a job are known to have a better health than individuals with a low SEP that do not work (Schuring et al., 2010), and who would not be part of the current sample. In addition, possibly the ‘healthier’ employees participated in the studies and the employees with the largest health potential were not reached (Dieker et al., 2019; Persson et al., 2013). Future research on workplace health promotion should focus on the challenge of attracting also those employees with the largest health potential. Participative approaches have been recommended before (WHO, n.d.), in which employees are involved in the development of workplace health promotion so that programs are developed in such way that they are considered relevant and feasible by employees with a low SEP, also by those with a poorer health.

Next to that, future research might need to take into account other risk factors of poor self-perceived health, next to health behavior. Research among older workers has shown that working conditions such as physical job demands, job control and job rewards influence self-perceived health as well, in some cases even more than health behavior (Schram, 2020). Especially employees with a low SEP are generally faced with working conditions that can have an impact on health such as low job control and high physical demands (Lundberg et al., 2007). The challenge for future research is to develop workplace health promotion programs that combine the various risk factors of self-perceived health for employees with a low SEP in order to improve health. Integrated approaches that combine health protection focused on working conditions with health promotion are considered promising in this regard (Baron et al., 2014) and could therefore be explored.

## Methodological strengths and limitations

A strength of this meta-analysis in which only studies that were performed in the Netherlands were included, is that it allowed to compare different health promotion programs in a homogeneous occupational health context. In the Netherlands all employees have access to occupational health care through their employer, who is responsible for sickness benefits during the first two years of sickness absence. Because of this a specific context, a dataset including Dutch intervention studies only allowed to make a fair comparison.

A first limitation of this study is that there were relatively few studies that measured self-perceived health *and* consisted of employees with a low SEP. Also, participants with a low SEP were often limited in the eligible studies, which resulted in studies dropping out for analysis. However, it is doubtful if more data and thus power would have led to differential effects, because there was no (neither positive or negative) overall effect. A second limitation is the comparability between studies in terms of focus on employees with a low SEP. In two studies, the workplace health promotion programs were specifically targeted on employees with a low SEP (Groeneveld et al., 2010; Viester et al., 2018), while the programs in the other four studies were not focused on a specific SEP group. Possibly, the effects in our study would be different if all studies were targeting employees with a low SEP in particular. However, the effects reported in our forest plots as well as the sensitivity analysis did not show deviant effects in the studies that did focus on employees with a low SEP. A third possible limitation is that the large confidence intervals for the interaction effects for gender and marital status could indicate a power problem.

## Conclusion

This meta-analysis did not find evidence for an effect of workplace health promotion programs on self-perceived health of employees with a low SEP. Also no differential effects were found, indicating that the programs in their current form do not target the health potential of employees with a low SEP employees. Future research should focus on the determinants of self-perceived health next to health behavior to improve the health of employees with a low SEP.

## Ethics approval

The Medical Ethical Committee of Erasmus MC Rotterdam declared that the Medical Research Involving Human Subjects Act does not apply to the current IPD meta-analysis (MEC- 2018–1143). As existing data were used, no additional consent to participate was obtained.

## Submission declaration and verification

The work has not been published previously and is not under consideration elsewhere.

## Author contributions

The IPD dataset was created by PC, KOH and SR. Analyses were performed by PC and HvH. HvH drafted the paper, and all authors (CB, SR, KOH, JvB, EdV and PC) provided intellectual input and critically reviewed the manuscript. All authors reviewed and approved the final manuscript. PC is the study guarantor.

## Role of funding source

The study was funded by The Netherlands Organization for Health Research and Development (ZonMw; grant number 53-1001-411 and 50-53115-98-018). The funding source did not play a role in the research or preparation of the article.

## Declaration of competing interest

None.
